# Supplementary material for: The interactome of intact mitochondria by cross-linking mass spectrometry provides evidence for coexisting respiratory supercomplexes
Source: Mol Cell Proteomics. 2017 Dec 8;17(2):216–32. doi: 10.1074/mcp.RA117.000470 (PMC5795388; doi:10.1074/mcp.RA117.000470)
Supplement: Supplemental Data [file supp_RA117.000470_133922_0_supp_23978_fzffwf.zip › spectra_annotation/mito_spectra_annotation/31-1-2-1-7-1.pdf]

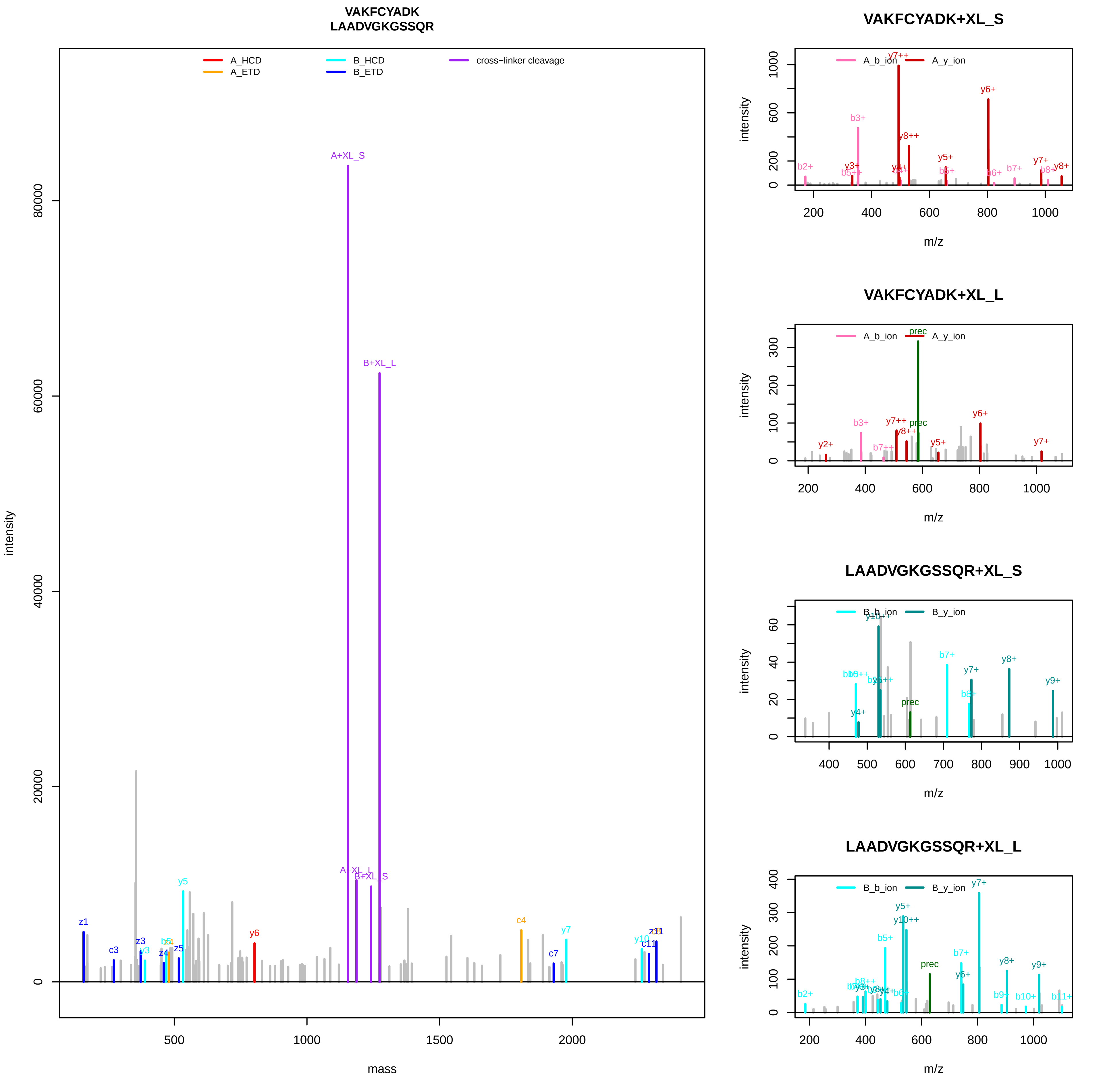
VAKFCYADK
LAADVGKGSSQR
A_HCD
A_ETD
B_HCD
B_ETD
cross−linker cleavage
A+XL_S
B+XL_L
A+XL_L
B+XL_S
z1
c3
z3
y3
z4
b5
c4
z5
y5
y6
c4
c7
y7
y10
c11
z11
intensity
mass
0
20000
40000
60000
80000
500
1000
1500
2000
VAKFCYADK+XL_S
A_b_ion
A_y_ion
y7++
y6+
b3+
y8++
b2+
y3+
b5++
y5+
y7+
y8+
b7+
m/z
VAKFCYADK+XL_L
prec
y2+
b3+
y7++
y8++
b7++
y5+
y6+
y7+
LAADVGKGSSQR+XL_S
B_b_ion
B_y_ion
y10++
y4+
b7+
y7+
y8+
y9+
prec
LAADVGKGSSQR+XL_L
y7+
y5+
y10++
b5+
prec
b7+
y6+
y8+
y9+
b2+
b10+
b11+
